# Public health management of invasive meningococcal disease in Baden-Wuerttemberg, Germany, 2012: adherence to guidance and estimation of resources required as determined in a survey of local health authorities

**DOI:** 10.1186/s12889-015-1693-6

**Published:** 2015-04-12

**Authors:** Lukas Murajda, Elisabeth Aichinger, Guenter Pfaff, Wiebke Hellenbrand

**Affiliations:** Baden-Wuerttemberg State Health Office, Stuttgart, Germany; European Programme for Intervention Epidemiology Training (EPIET), ECDC, Stockholm, Sweden; Robert Koch Institute, Berlin, Germany

**Keywords:** Sporadic invasive meningococcal disease, Public health management, Germany

## Abstract

**Background:**

Invasive meningococcal disease (IMD) incidence in Germany is low, but management of contacts to prevent subsequent cases still requires resources. Local public health authorities (LHA) advise antibiotic post-exposure prophylaxis (PEP) and vaccination to close contacts as defined in national guidance. We aimed to audit implementation of recommendations for IMD public health management in the state of Baden-Wuerttemberg, Germany, and to estimate associated costs.

**Methods:**

We surveyed all 38 LHAs in Baden-Wuerttemberg to evaluate knowledge of national guidance and implementation of IMD contact management using standardized questionnaires. For IMD cases notified in 2012, we requested numbers of household and other contacts ascertained, including advice given regarding PEP and post-exposure vaccination, plus staff time required for their management. We estimated costs for advised antibiotics, LHA staff time and visits to emergency departments according to published sources. The cost of preventing a subsequent case was estimated based on the number of household contacts that received PEP per IMD case and on the previous finding that ~284 household contacts must receive PEP to prevent one subsequent IMD case.

**Results:**

Although LHAs were familiar with national recommendations, they did not advise PEP to 4% of household contacts, while 72% and 100% of school and health provider contacts, respectively, were advised PEP. Only 25% of household contacts of a case with a vaccine-preventable serogroup were advised post-exposure vaccination. A mean of 11.0 contacts/IMD case (range 0–51), of which 3.6 were household contacts, were recommended PEP. Per IMD case, mean costs for LHA staff were estimated at €440.86, for antibiotics at €219.14 and for emergency department visits to obtain PEP at €161.70 - a total of €821.17/IMD case. Preventing a subsequent IMD case would cost ~ €65,000.

**Conclusions:**

Our results provide insight into costs of IMD public health management in Germany. We identified marked underuse of post-exposure vaccination in household contacts and overuse of PEP in school and health care contacts. In view of an estimated 3–6 quality-adjusted life years lost per case of IMD, our estimated cost of €65,000 for preventing a subsequent case seems justifiable.

## Background

Invasive meningococcal disease (IMD), caused by *Neisseria meningitidis*, manifests most commonly as meningitis or sepsis [1]. The incidence in Germany decreased in recent years, from 0.95 to 0.44 IMD cases/100,000 inhabitants, and similar decreases were observed in other European countries [2]. Nonetheless, even a single case can generate substantial public concern [3] because of high case fatality, propensity to affect the young and occasional appearance in clusters [4]. Modelling studies [5,6] estimated that 3–6 quality adjusted life years could be gained per case averted. Provision of antibiotic post-exposure prophylaxis (PEP) and, in case of a vaccine-preventable strain in the index case, post-exposure vaccination of defined contacts, are evidence-based measures to reduce the risk of subsequent IMD cases [7]. Household contacts of persons with IMD have a higher risk of acquiring the disease [8,9]. This also holds for close contacts in preschool settings, albeit to a lesser extent [10]. For household settings, the European Centre for Disease Prevention and Control (ECDC) estimated the number of contacts needed to be treated (NNT) with PEP to prevent one case at 284 (95% confidence interval (CI): 156–1515) [7]. For pre-school settings, NNT was estimated at 1998 (95% CI: 1307–4259). Much larger numbers of contacts would require treatment in school and university settings to prevent one IMD case [7]. By far, PEP provided to household contacts, who face the highest risk, contributes most to the prevention of subsequent cases. According to guidance of Standing Committee on vaccination and the Robert Koch-Institute (RKI) [11,12], the following persons are defined as close contacts of an IMD index case: 1) all household members, 2) contacts in the same group of an index case in kindergarten/pre-schools with children under six years, 3) household-like contacts, such as persons living in the same dormitory or military barracks as an index case, 4) persons with contact to oropharyngeal secretions of an index case, e.g. sexual partner, close friends, school contacts seated next to an index case, 5) medical staff if exposed to oropharyngeal secretions of an index case, e.g. through mouth-to-mouth resuscitation or unprotected intubation.

In Germany, IMD is statutorily notifiable by both physicians and laboratories. Cases are notified to local public health authorities (LHA) and forwarded to the state public health authorities according to a standardized case definition [13]. Surveillance data suggest that the case fatality of IMD is 8% [14].

The German Protection Against Infection Act requires local health authorities to implement measures to prevent further transmission of infectious diseases [15]. LHAs trace and manage contacts of cases with IMD according to guidance provided by the national public health institute, the Robert Koch Institute (RKI) [12], based on STIKO recommendations [11]. STIKO recommends PEP with an antibiotic that eradicates meningococcal carriage for close contacts of an IMD index case. Recommended antibiotics for children are rifampicin (20 mg/kg/b.i.d. for 2 days except 10 mg/kg/ b.i.d. for 2 days for newborns) or intramuscular (i.m.) ceftriaxone (125 mg. for < 12 year-olds, 250 mg i.m. for ≥12 year-olds), ciprofloxacin (500 mg single dose) or rifampicin (600 mg bi.d. for 2 days) for adults and ceftriaxone (250 mg i.m.) for pregnant women [11]. Post-exposure vaccination is recommended for household (-like) contacts if the IMD index case is diagnosed with a vaccine-preventable serogroup (in 2012: A, C, W, Y). Guidance on management of invasive meningococcal disease is also made available by the National Reference Laboratory for Meningococci in Wuerzburg [16], the German Society for Paediatric Infectious Diseases [17], the Working Group Meningococci of the German Green Cross for Health [18] and in a handbook on infectious diseases by Littmann et al. [19]. In December 2013, a new vaccine licensed to protect against serogroup B meningococci was made available on the European market. RKI and STIKO planned to model the impact of implementation of this meningococcal B vaccine. However, evidence on costs of public health management of sporadic IMD in Germany or other countries was lacking at the time this study was launched. A systematic review was available on the cost of managing IMD outbreaks [20], including implementation of chemoprophylaxis and targeted vaccination. Costs ranged from $2,000-56,000 (≈€1,600-45,000) per one IMD case [20], depending on the size and location (high or low income country) of the outbreaks. We performed an audit of the implementation of national recommendations for public health management of IMD in the federal state of Baden-Wuerttemberg, Southwest Germany, with a population of 10.5 million. Our objective was to evaluate knowledge of national guidance in LHAs and to describe actual implementation of management of contacts of sporadic IMD to estimate associated costs.

## Methods

### Cross-sectional study on knowledge and awareness of national recommendations for IMD contact management

In May 2013, we surveyed head physicians of all 38 LHAs in Baden-Wuerttemberg regarding usual practices applied in each LHA for public health management of notified IMD cases. We sent a questionnaire to collect information on use of available guidance documents for public health management of IMD, criteria applied to define and identify contact persons for PEP or post exposure vaccination and the number of staff available for public health management of infectious diseases.

### Retrospective case study on costs associated with IMD contact management

In a second questionnaire, we asked the head physicians of LHAs that had notified one or more IMD cases in 2012 to provide information on the number and types of contacts identified for each case notified in 2012, and whether PEP or post-exposure vaccination had been advised. In addition, the questionnaire collected information on the number and working hours of physicians and other staff involved in the management of each notified case.

### Cost identification

We used the results from the cross sectional study to identify the antibiotics advised by LHAs for PEP in children, adults and pregnant women. We estimated the proportion of pregnant women among adult contact persons based on population census and live births data for Baden-Wuerttemberg [21].

### Medications

We estimated the cost of antibiotics advised to contacts of notified cases using the German medical formulary for 2014 [22]. We used the price for the smallest unit provided by the manufacturer – the lowest possible number of tablets or the lowest possible volume of oral liquid formula purchasable and sufficient to treat one person. The cost of the smallest units of antibiotics recommended for PEP in tablet form were as follows: €39.47 for blister of ten splittable 600 mg rifampicin tablets, €14.36 for a blister of fourteen 500 mg ciprofloxacin tablets and €5.36 for a single i.m. dose of ceftriaxone. The cost of the smallest unit of rifampicin syrup, 100 ml containing 1.2 g of rifampicin, was €21.60.

### Health care delivery

As physicians can bill for only one patient visit per quarter (i.e. 4 times per year), we did not include costs for office visits to obtain PEP. We assumed that most contacts would see their doctor in the current quarter for other reasons. However, we included visits to emergency services, estimated at €30 each according to a survey of emergency departments [23].

### Staff cost

We estimated the cost of full-time equivalent (FTE) staff involved in management of the contacts based on remuneration according to the civil service pay scale, as published by the Association of Municipal Employer associations (VKA) in 2014 [24]. We calculated daily wages based on annual wages assuming 230 working days/year. We assumed that LHA physicians were paid according to the highest grade of remuneration at level 15 (€290.81/day), hygiene inspectors according to the highest grade of remuneration at level 8 (€153.11/day) and administrative staff according to the highest grade of remuneration at level 6 (€140.59/day).

We estimated the number of household contacts who received PEP per one IMD index case (HC). Taking into consideration the known NNT (284) to prevent one subsequent case in household settings, contact management of N cases (N = 284/HC) potentially prevented one subsequent case in household settings. We thus estimated the approximate cost of preventing one subsequent IMD case in Baden-Wuerttemberg through public health management as N*(FTE staff cost + cost of antibiotics + cost of visit at emergency services).

### Data analysis

We entered data in EpiData Entry 3.1. (EpiData Association, Odense, Denmark). Analysis was performed using Excel (Excel 2007, Microsoft Corporation, US) and Stata 12 (Stata Corp, Texas, US). Denominators vary due to missing responses on the questionnaires.

### Ethical approval

The study was based on statutory case notifications of meningococcal disease to public health authorities in the federal State of Baden-Wuerttemberg for the year 2012, as mandated by the German Protection against Infection Act. These data were available in anonymized form at the federal state and national levels, i.e. information concerning personal or material circumstances could thus no longer or only with an incommensurate amount of effort, time and expense be linked to an identified or identifiable individual. Ethical approval for analysis of such surveillance data is not required according to the Medical Association’s professional code of conduct [25]. The additional data collected in relation to these cases at the level of the local health authorities did not involve further information on the patients themselves, but only aggregated information on their contact management and associated resources required at the level of the local health authorities, i.e. the legal status of the data as anonymized remained unchanged. Thus ethical approval for performance of this study was not considered necessary.

## Results

### Implementation of national recommendations for IMD contact management

Of the 38 LHA, 34 returned the questionnaire (response 89%). All responding LHAs stated they followed national recommendations to manage IMD contacts. LHAs most frequently used documents issued by RKI (32/34, 94%) or STIKO (27/33, 82%), with only one LHA using neither of these. Three LHAs also used recommendations issued by the National Reference Laboratory for Meningococci in Wuerzburg, and one each used recommendations published by the German Society for Paediatric Infectious Diseases, the Working Group Meningococci of the German Green Cross for Health. Two LHAs also used their own guidelines and one LHA used a handbook [19].

In line with RKI/STIKO guidance, all LHAs stated they would advise PEP to all household members of an IMD case (Table 1). However, 29% of LHAs reported that they would advise PEP to more contacts than recommended by RKI/STIKO in kindergarten/preschool settings, 47% in schools and 32% in healthcare settings (Table 1).

**Table 1:** **Invasive meningococcal disease (IMD) contacts to whom local health authority (LHA) would recommend antibiotic post-exposure chemoprophylaxis (PEP)**

**Contact category**		**No. LHAs that advised PEP**	**Total**	**%**
Household	All household members	34	34	100
Kindergarten/preschool	All children in the facility	10	34	29
	Only the group of index case	23	33	70
	Teacher of the index case	31	32	97
School	Only pupils sitting in neighbouring seat	17	32	53
	Class of the index case	15	32	47
	Teacher of the index case	17	32	53
Students’ residence or similar (e.g. barracks, hotels)	All residents	1	34	3
	Only residents on the same floor	13	34	38
	Only residents in the same room	20	34	59
Travellers*	Contact with oropharyngeal secretions of index case	33	33	100
	Sitting in proximity to index case	31	32	97
	Contact longer than a specified time**	9	30	30
Healthcare workers	Any contact with index case	11	34	32
	Only if contact with oropharyngeal secretions of index case	22	34	65

Survey of 34 Local Health Authorities (LHA), Baden-Wuerttemberg, Germany, 2013.

*Travellers = person travelled in the same airplane, train or bus as the index case.

**Time interval specified between one and eight hours.

Of the several antibiotics that LHAs stated advising for PEP in different age groups, rifampicin was most often advised for infants, children and adolescents, ciprofloxacin for adults, and ceftriaxone i.m. for pregnant women (Table 2). In terms of the mean number of full time equivalent (FTE) staff positions available at epidemiology departments for public health management of infectious diseases, the 34 responding LHAs reported 1.6 physicians (range 0.75-3.5), 2.3 hygiene inspectors (range 0–8) and 1.2 administrative staff (range 0–3).

**Table 2:** **Antibiotics recommended by local health authority (LHA) for post-exposure prophylaxis of different categories of invasive meningococcal disease contacts, Survey of 34 LHA, Baden-Wuerttemberg, Germany, 2013**

	**LHAs recommending antibiotic**											
	**Rifampicin**			**Ciprofloxacin**			**Ceftriaxone i.m.**			**Other [which]**		
**Contact category**	**n**	**N**	**%**	**n**	**N**	**%**	**n**	**N**	**%**	**n**	**N**	**%**
**Infants**	28	29	97	1	29	3	-	-	-	1	30	3 [unspecified]
**Toddlers**	30	30	100	1*	30	3	2*	30	7	-	-	-
**School children (5–11 years)**	30	30	100	5*	30	17	-	-	-	-	-	-
**Adolescents (12–17 years)**	28	30	93	4**	30	13	7*	30	23	-	-	-
**Adults**	16^†^	30	53	27	30	90	3‡	30	10	-	-	-
**Pregnant women**	1	30	3	1	30	3	27	30	90	1	30‡‡	3 [azithromycin]

n = number of LHAs recommending this antibiotic; N = total number of LHAs responding to question; *in addition to rifampicin; **2/4 in addition to rifampicin; †13/16 in addition to ciprofloxacin, thus 3 (10%) recommended only rifampicin; ‡ in addition to ciprofloxacin; ‡‡in addition to ceftriaxone.

### IMD contact management

In 2012, 27 LHAs notified 49 IMD cases. Twenty-three LHAs returned questionnaires on 41 IMD cases (84% response).

The responding LHAs identified 473 contact persons for these 41 cases, of whom 451 (95%, 324 adults, 127 children) were advised to take PEP for a mean of 11 contacts per index case of IMD (range: 0–51, 3 children and 8 adults). Of these 451 contacts, 148 (33%) were household members of 33 cases, 83 (18%) medical personnel of 14 cases, 34 (8%) school children of 4 cases, 3 (0.7%) fellow residents in a dormitory of 1 case, 73 (16%) family visitors of 14 cases and 110 (24%) other contacts of 20 cases (Table 3). As age was not specified for family visitors and other contacts, we assumed 50% each were adults and children < 18 years. The latter consisted mainly of work colleagues and friends, including contacts at a disco. No case had contacts in a kindergarten/preschool setting. The proportion of ascertained contacts advised to obtain PEP was high, at 72% in school contacts and over 95% in all other categories (Table 3).

**Table 3:** **Number of contacts and proportion advised post-exposure prophylaxis as reported by local health authorities (LHA) for 41 invasive meningococcal disease (IMD) cases notified in 2012 in Baden-Wuerttemberg, Germany**

**Category of IMD contact**	**No. contacts recommended PEP**	**No. cases with ≥1 contact**	**Contacts per case**		**% contacts recommended PEP**
			**Mean**	**Range**	
**Household members 0–18 years**	35	16	0.9	0-9	97
**Household members >18 years**	113	35	2.9	0-15	96
**Healthcare workers**	83	14	2.0	0-21	100
**Kindergarten/preschool**	NA	0	0	-	NA
**School**	34	3	1.1	0-21	72
**Dormitory or similar**	3	1	0.07	0-3	100
Family visit*	73	14	1.8	0-17	100
**Children (<19 years)**	37	14	0.9	-	100
**Adults (>18 years)**	36	14	0.9	-	100
**Other contacts**	110	20	2.8	0-42	97
**Children (<19 years)**	21	4	0.4	0-10	100
**Adults (>18 years)**	89	16	2.2	0-42	100

A total of 451 contacts, 127 children and 324 adults, were recommended PEP.

NA: Not applicable *As age was not specified, we assumed ~50% each children and adults.

Of the 41 IMD cases for whom a questionnaire was returned, 12 were caused by a vaccine-preventable serogroup (8 C, 1 W and 3 Y), 26 were caused by serogroup B and the serogroup was unknown in 3 cases. The 12 cases with a vaccine-preventable serogroup had a total of 36 household contacts, 9 (25%) of whom were advised to obtain post-exposure vaccination. Of the 95 household contacts of cases with the then non-vaccine-preventable serogroup B, 6 (6.3%) were nonetheless advised to obtain post-exposure vaccination, including one adolescent. In addition, 20 non-household contacts were advised to receive post-exposure vaccination, 15 of whom were contacts of a case with a vaccine-preventable serogroup. Three persons were living in the same dormitory as the case, 8 were medical staff, 1 was a school contact and 3 were other contacts.

LHA reported that contacts obtained antibiotics for PEP either through a general practitioner (GP) (all 33contacts of 5 cases), from a hospital (all 22 contacts of 7 cases) or, in 27 cases, from a combination of both supply channels (417 contacts). We assumed that half of the latter obtained PEP through each of these supply channels, respectively. Some contacts of 2 cases received antibiotics directly from the LHA.

### Costs of IMD contact management

Management of a notified case of sporadic IMD mobilized the majority of physicians, hygiene inspectors and other administrative personnel assigned to public health management of infectious diseases at LHAs for 1–2 days (Table 4). The cost of work of staff at LHAs per one IMD case amounted €343 for physicians, €54 for hygiene inspectors and €44 for administrative staff as derived in Table 4. The overall estimated total staff cost was €441 per IMD case.

**Table 4:** **Staff time required for public health management of an invasive meningococcal disease (IMD) case, survey of 34 local health authorities (LHA), Baden-Wuerttemberg, Germany, 2013**

	FTE* staff				
	**Mean**	**Range**	**Mean person-days**	**Person-days (range)**	**Staff cost**
**Physicians**	1.58	1-3	1.18	0.12-3.00	€343
**Hygiene inspectors**	1.00	0-5	0.35	0.12-2.50	€54
**Other personnel**	1.26	0-2	0.31	0-2.00	€44
**Total**	3.84		1.84		€441

*FTE = full-time equivalent.

Based on the cross-sectional survey, for adults, 10% of LHAs advised exclusively rifampicin, 47% exclusively ciprofloxacin and 43% either rifampicin or ciprofloxacin (Table 2). As a single-dose regimen of ciprofloxacin is likely perceived as more convenient, we estimated that only a minority - 20% - of adult contacts received rifampicin, 79% of adults contacts received ciprofloxacin and 1% of adult contacts (pregnant women) received ceftriaxone i.m. We simplified cost estimation for children by assuming that all children received 100 ml (1.2 g) rifampicin syrup. For children > 30 kg (≈ > 10 years of age), who would require a higher dosage, we nonetheless applied the lower price for 1.2 g of the rifampicin syrup, under the assumption that some children would not receive a separate prescription, but share PEP antibiotics from a unit prescribed to their parents, and a small (but unknown) proportion of older children would be prescribed the lower-priced ciprofloxacin.

Since for each notified IMD case the LHAs advised PEP to a mean of 3 children and 8 adults, the cost of PEP per one IMD case was €219 (Table 5).

**Table 5:** **Estimated average cost of antibiotics for contacts of one invasive meningococcal disease (IMD) case, survey of 34 local health authorities (LHA), Baden-Wuerttemberg, Germany, 2013**

**IMD Contact persons**	**Antibiotics used**	**Cost of antibiotics (€)**	**Percent of contacts**	**Cost per average contact**	**No. of contacts per IMD case**	**Total cost per case**
Children	Rifampicin syrup	21.60	100%	21.60	3	64.80
Adults	Rifampicin tablets	39.47	20%	19.29	8	154.34
	Ciprofloxacin tablets	14.36	79%			
	Ceftriaxone i.m.	5.36	1%			
Total						219.14

According to LHA, 49% of contacts received PEP through emergency services, translating to 5.39 contacts per case. The cost per one IMD case was thus €162 (= 5.39 *€30).

We thus estimated the total cost of managing the contacts of an IMD case at LHAs at €822 (€441 for personnel + €219 for antibiotics + €162 to obtain PEP through emergency services).

In our survey, for each IMD index case, 3.6 household contacts and 7.4 other contacts received PEP. Taking into account the NNT for household contacts [7], contact management in Baden-Wuerttemberg prevented one subsequent case in household settings at a cost of ≈ €65,000 ((284/3.6)*€822).

We made a rough estimate of the cost of recommending PEP unnecessarily to contacts in the school and health care setting, although this was limited by the low number of cases with school contacts. We assumed that in general no more than 2 school or health care contacts should receive PEP according to recommendations. In our study, in 2 cases, 6 and 21 school contacts, and in 10 cases, >2 health care contacts ( a total of 78 contacts) were recommended PEP, translating to potentially 23 and 58 contacts, respectively, with an unnecessary recommendation. Subtracting these numbers from the total number of child and adult contacts advised to obtain PEP according to our survey decreased the number of child and adult contacts requiring PEP to 2.5 (from 3.0) and 6.5 (from 8.0) per case, respectively, reducing the cost estimate per case for antibiotics from €219 to €179 and the costs for emergency services from €162 to €133. The total costs per case would thus be reduced to €753, and the cost for preventing a subsequent case to ≈ €59,000.

## Discussion

Our results indicated that LHAs in Baden-Wuerttemberg were aware of the available national recommendations on public health management of IMD. Almost all used RKI/STIKO guidance documents. Antibiotics for PEP advised by LHAs were all in keeping with this guidance. However, according to the cross-sectional survey, LHAs tended to advise PEP to a wider circle of contacts than recommended in the national recommendations. The retrospective case study also suggested that a substantial number of contacts without an indication received antibiotics, as LHAs advised PEP for 34 of the 47 school contacts and for all 83 health care worker contacts. On the other hand, a few household contacts (4%) were not advised to obtain antibiotics, despite all LHAs having stated that they would, in principle, advise PEP to all of these.

Only a quarter of household contacts of an index case with a vaccine-preventable serogroup were advised to receive post-exposure vaccination. A possible explanation for this is that the serogroup may not be known at the time of notification for some cases. This delay in the availability of information would make further contact necessary to advise vaccination once serogroup information becomes available. On the other hand, post-exposure vaccination was advised to a number of household contacts even though the index case was not due to a vaccine-preventable serogroup. One such contact was of adolescent age for which meningococcal C vaccine is generally recommended. Some contacts, most notably health care workers, were advised post-exposure vaccination even though this was not recommended according to RKI/STIKO guidance. A high level of anxiety upon the occurrence of IMD cases and a desire to do everything possible to prevent further occurrence of this severe disease, as well as pressure from the contacts themselves, particularly health care workers, may explain the tendency to recommend PEP or vaccination to more contacts than necessary.

Our results indicated that management of the contacts of an IMD case engaged most members of the LHA infectious disease public health team for 1–2 days, thus putting a considerable strain on the staff and likely requiring postponement of other public health tasks.

We estimated the cost of preventing a subsequent case of IMD through public health management of a sporadic cases of IMD to be ≈ €65,000. Since ≈ 3-6 QALYs are lost through a case of IMD [5,6], this amounts to ≈ €11,000-22,000 per QALY gained, or ≈ €10,000-20,000 if PEP were recommended more judiciously. While the use of cost-effectiveness thresholds is controversial and has not been applied for medical or preventive interventions in Germany thus far, threshold values applied in various countries or by certain institutions in the past considered to be cost-effective generally ranged from $20,000 to $100,000 (≈€15,000-80,000) per QALY [26]. Thus, the seemingly high cost of preventing one IMD case of €65,000 through contact management according to our study appears justifiable - also in view of the severity, high case fatality, and the high treatment costs for acute disease, complications and sequelae in a high proportion of cases [27,28]. Furthermore, the cost per IMD case prevented could be reduced if LHAs limited their advice for PEP to contacts according to national recommendations.

Our estimate of resources required for public health management of IMD cases has several limitations. First, it was based on only 41 cases of IMD from only one federal state in Germany and these do not mirror the entire range of possible contact scenarios. For instance, none of the contacts were infants or children in kindergarten/preschool. In addition, some contact scenarios applied only to a very low number of cases. Second, we may have overestimated the cost for antibiotics, as in rare cases contacts might receive single doses directly in the emergency department rather than the smallest available blister pack. However, as we chose the lowest possible price of the smallest possible quantities, it is also possible that costs were higher for some individuals. In addition, we did not include costs for any doctors’ office visits to obtain PEP, although in some cases they may have applied. Finally, we did not include the costs of post-exposure vaccination. Overall, these limitations would have led us to under-estimate the costs for obtaining PEP. Nonetheless, our results provide insight into the magnitude of costs for public health management of sporadic IMD in Germany. Since termination of our study, a British study estimated a somewhat lower cost for the public health management of a single IMD case at £317.72 (approximately €400) [29]. However, this was based on an analysis of a single index case in a cluster of two cases in a primary school. Costs for management of the two epidemiologically linked cases were 17-fold higher than management of a primary case, in keeping with the high costs found for management of IMD clusters in [20].

## Conclusions

The awareness of national recommendations for public health management of IMD was high in LHAs of Baden-Wuerttemberg. However, LHAs advised PEP to a wider circle of contacts than specified in the recommendations. At the same time, implementation of post-exposure vaccination among household contacts with a vaccine-preventable serogroup was markedly underused. To close this identified knowledge-practice gap, we encouraged LHAs to limit advice for PEP to contacts as defined in national recommendations and to improve implementation of post-exposure vaccination for household contacts. A better implementation of the national recommendations would optimize prevention and avoid unnecessary expenses. Finally, our results provide insight into the costs of public health management in Germany for the first time and are being used to parameterize a model to estimate the costs and benefits of meningococcal B vaccination in Germany.
